# Drug Reaction With Eosinophilia and Systemic Symptoms (DRESS) Syndrome Caused by Pola-R-CHOP: A Case Report

**DOI:** 10.7759/cureus.94972

**Published:** 2025-10-20

**Authors:** Mohammed Aamir, Aamir Mohamed Nur

**Affiliations:** 1 Haematology, University Hospitals of Leicester, Leicester, GBR

**Keywords:** cytomegalovirus-cmv, diffuse large b cell lymphoma (dlbcl), drug reaction with eosinophilia and systemic symptoms (dress) syndrome, pola rchop, systemic steroids

## Abstract

Chemotherapy regimens, especially for haematologic malignancies, have been increasingly implicated in drug reactions with eosinophilia and systemic symptoms (DRESS) syndrome. We present a case of DRESS syndrome in a patient with diffuse large B-cell lymphoma (DLBCL) receiving pola-R-CHOP chemotherapy, highlighting the importance of early recognition and management. A 58-year-old woman with DLBCL, previously treated breast cancer, and other comorbidities presented three weeks after her third pola-R-CHOP cycle with fever, hypotension, lethargy, and a worsening rash. Initially suspected of peripherally inserted central catheter (PICC) line infection, she was admitted to the ICU for vasopressor support and had acute renal dysfunction. Examination revealed a widespread erythematous, purpuric rash with superficial scaling, lip erosions, and mild conjunctival involvement. Laboratory findings showed eosinophilia and elevated ALT. Despite atypically low eosinophil counts, the rash's timing, systemic symptoms, and organ involvement raised suspicion for DRESS syndrome. The patient also had a history of CMV reactivation and was on ganciclovir. However, the clinical presentation favoured DRESS as the primary diagnosis. Initial treatment included empirical antibiotics and vasopressors. With infection ruled out, IV hydrocortisone was started, followed by a switch to oral prednisolone (1 mg/kg), leading to clinical improvement. Due to a positive CMV PCR, the steroid dose was reduced, resulting in clinical deterioration, increased eosinophils, and worsening liver/renal function. Prednisolone was increased to 80 mg with subsequent improvement, then tapered gradually as per dermatology advice. The patient was discharged on a structured steroid weaning plan and scheduled for close outpatient monitoring. This case highlights the diagnostic challenge of DRESS in immunocompromised oncology patients, particularly when CMV reactivation complicates the picture. Though eosinophilia is a hallmark of DRESS, it may be mild, and clinicians should not exclude the diagnosis based solely on counts. The latency period post-chemotherapy, classic dermatologic features, and systemic organ involvement aligned with DRESS. Prompt withdrawal of the causative agent and initiation of corticosteroids are key to preventing progression to multi-organ failure. This case report highlights that DRESS syndrome, although rare, should be part of the differential in oncology patients receiving multi-agent therapies who present with systemic symptoms and rash. Early identification and management are crucial to reducing morbidity and mortality.

## Introduction

A 58-year-old female with diffuse large B-cell lymphoma on pola-R-CHOP chemotherapy presented with fever, hypotension, and a progressive rash three weeks after her last treatment cycle. Initially suspected of having a PICC line infection, she required ICU admission for vasopressor support and had acute renal dysfunction. Clinical findings included a widespread erythematous, purpuric rash with superficial scaling, lip erosions, and mild conjunctival involvement. Laboratory results showed eosinophilia and raised ALT. Given the temporal association with chemotherapy and systemic involvement, DRESS syndrome was suspected despite atypical eosinophil counts. Prior CMV reactivation and current ganciclovir treatment were noted. The case underscores the importance of early recognition and prompt withdrawal of the causative drug in suspected DRESS to prevent multi-organ failure and reduce mortality. Clinicians must maintain a high index of suspicion in oncology patients receiving multi-agent therapies, especially when systemic symptoms and dermatologic findings coincide.

DRESS syndrome (drug reaction with eosinophilia and systemic symptoms) is a rare but serious adverse drug reaction that can be life-threatening if not promptly recognized and treated. It typically occurs 2-8 weeks after the initiation of certain medications, including anticonvulsants, sulphonamides, and allopurinol [1]. The syndrome is characterized by a combination of skin rashes, fever, elevated eosinophil counts, and multi-organ involvement, particularly affecting the liver, kidneys, and lungs. In severe cases, it can lead to multi-organ failure, which is why early diagnosis is critical [2]. While rare, DRESS syndrome has become more prominent in clinical practice due to the growing use of polypharmacy, particularly in patients with chronic conditions, making it an increasingly relevant health issue [3].

The pathophysiology of DRESS syndrome is immune-mediated and involves complex interactions between genetic factors and drug-induced immune responses. In genetically susceptible individuals, drugs act as haptens, binding to host proteins and forming a complex that triggers a hypersensitivity reaction. This activates T-cells and eosinophils, leading to widespread inflammation in multiple organs. The immune response is marked by the release of cytokines and the infiltration of eosinophils, which contribute to the systemic symptoms [4]. Genetic predispositions, including specific human leukocyte antigen (HLA) alleles, can increase susceptibility to this reaction. The key takeaway for clinicians is that early recognition and prompt withdrawal of the causative drug are essential for improving patient outcomes, preventing severe complications, and reducing mortality [5].

## Case presentation

A 58-year-old female with a medical history of diffuse large B-cell lymphoma (DLBCL), breast cancer (mastectomy 2015), hypertension, and osteoarthritis was receiving chemotherapy with the pola-R-CHOP regimen. She had completed three cycles of chemotherapy, with the most recent cycle administered in June 2025.

The patient was admitted on 22nd July 2025 with complaints of fever, lethargy, hypotension, and a progressively worsening rash. She was initially suspected to have a PICC line infection due to her hypotension and fever, leading to transfer to the intensive care unit (ICU) for vasopressor support. She also had acute renal dysfunction, which exacerbated her clinical deterioration. Bloods on admission are shown in Table 1.

**Table 1:** Laboratory investigations on admission

Investigation	Result	Reference ranges
White cell count	11.9	4-11 x 10⁹/L
Neutrophil count	10.32	1.5-7.5 x 10⁹/L
Eosinophil count	1	0.04-0.4 x 10⁹/L
Platelet count	84	140-400 x 10⁹/L
Lymphocyte count	0.23	1-4 x 10⁹/L
Alanine Transaminase (ALT)	396	10-49 U/L
Bilirubin	22	0-21 mcmol/L
Creatinine	240	60-120 mcmol/L
C-Reactive Protein (CRP)	166	0-10 mg/L

Three weeks prior to admission, she developed a maculopapular rash, which progressed to areas of purpura and superficial scaling. Lip erosions and perioral crusting were noted. The patient also complained of mild conjunctival irritation with a crusted discharge from her eyelashes. There was no genital mucosal involvement, and she denied any significant oral pain or new oral lesions initially.

Upon examination, the patient was alert and clinically stable, but the skin rash had become extensive, involving the trunk, arms, and thighs. The rash was erythematous, with areas of purpura suggestive of possible vasculitis. There were superficial scaling lesions on the upper back, chest, and forearms. Mild facial swelling and crusting on the lips were present, with a negative Nikolsky’s sign and no new blistering, apart from a small blister on the left big toe, which was thought to be due to bed friction. Mild conjunctival crusting and lip erosions were also observed.

The patient had a history of CMV reactivation with a positive CMV PCR in May and June 2025 and was currently being treated with ganciclovir for this. The presence of raised eosinophils, ALT, and the temporal onset of the rash in relation to chemotherapy strongly suggested DRESS syndrome. Although eosinophil counts were not as high as those in typical DRESS cases, the combination of skin and mucosal involvement, fever, and systemic inflammation raised concern for this life-threatening condition.

A comprehensive differential diagnosis list was undertaken, which included DRESS syndrome (drug reaction with eosinophilia and systemic symptoms), a severe hypersensitivity reaction that typically occurs 2-8 weeks after exposure to a triggering drug. It is characterized by skin rashes, eosinophilia, fever, and involvement of internal organs such as the liver and kidneys. Common triggers include anticonvulsants, antibiotics, and chemotherapeutic agents. This patient's presentation with a progressive maculopapular rash, eosinophilia, and elevated liver enzymes (ALT) closely aligned with DRESS syndrome. The temporal relationship between the third cycle of chemotherapy (pola-R-CHOP ) and the rash, along with eosinophilia, fever, and liver dysfunction, pointed towards DRESS as the most likely diagnosis. The patient also developed mucosal erosions and perioral crusting, which are common features of DRESS. Additionally, the elevated eosinophil count and renal dysfunction also supported this diagnosis.

CMV reactivation (cytomegalovirus reactivation) was also considered, given the patient's history of CMV infection and recent positive CMV PCR results. CMV reactivation in immunocompromised patients, such as those undergoing chemotherapy, can cause a variety of symptoms, including fever, mucosal ulcerations, and organ dysfunction. The patient was positive for CMV DNA PCR from mouth and lip swabs and had previously been treated for CMV. However, CMV reactivation typically does not present with the same eosinophilic rash or the extensive skin changes seen in this patient. Additionally, there was no worsening of symptoms with ganciclovir, which makes CMV less likely as the primary cause of her current presentation. CMV-associated rashes are less common and usually not as prominent as those seen in DRESS syndrome.

SJS/TEN (Stevens-Johnson Syndrome/Toxic Epidermal Necrolysis) is another severe drug-induced skin reaction characterized by extensive skin detachment, mucosal ulcerations, and systemic involvement. These conditions are usually caused by medications such as antibiotics, anticonvulsants, and nonsteroidal anti-inflammatory drugs (NSAIDs). The mucosal ulceration seen in this patient, along with the rash, raised initial concern for SJS/TEN. However, the lack of widespread blistering, epidermal detachment, and Nikolsky’s sign being negative made SJS/TEN less likely. This, coupled with the progressive rash and eosinophilia, suggested that DRESS syndrome was a more likely diagnosis.

Infective causes, particularly a PICC line infection, were considered due to the patient’s fever, hypotension, and history of PICC line insertion for chemotherapy. PICC line infections can present with fever and sepsis-like symptoms, and they are known to cause skin changes. However, blood cultures were negative for any bacterial growth, and the patient’s rash was more immune-mediated in nature rather than being infectious. The eosinophilia and skin lesions in this case were more suggestive of a drug-induced reaction, further ruling out an infective cause.

In conclusion, after careful consideration of these differentials, the diagnosis of DRESS syndrome was confirmed, likely triggered by the chemotherapy and possibly co-trimoxazole and acyclovir. This was supported by the temporal relationship between chemotherapy and symptom onset, the characteristic skin rash, eosinophilia, liver dysfunction, and the improvement with steroid therapy. While CMV reactivation and SJS/TEN were considered, the absence of specific signs for these conditions led to a final diagnosis of DRESS syndrome.

Initial management focused on addressing potential sepsis from a suspected PICC line infection. The patient was started on empirical antibiotics, including Tazocin and vancomycin, and received vasopressor support for persistent hypotension unresponsive to IV fluids. A full septic screen was conducted by the ICU team, which ruled out infection as the primary cause of her deterioration. Given the clinical features, including severe skin changes and systemic symptoms, DRESS syndrome was suspected, and the patient was started on IV hydrocortisone. Dermatology was consulted for ongoing management, and after review, the IV hydrocortisone was switched to oral prednisolone at a dose of 1 mg/kg. Over time, the patient’s condition improved, and she was transferred from the ICU to the ward.

However, CMV DNA PCR from both blood plasma and lip/mouth swabs returned positive, prompting a reduction in the prednisolone dose from 80 mg to 40 mg. The patient acutely deteriorated, presenting with a NEWS score of 11, fever (40°C), tachycardia, and hypotension (blood pressure in the 60s). Her eosinophil count, which had previously improved, increased again to 1.39, and her ALT and renal function worsened, as shown in Table 2. As a result, the prednisolone dose was increased back to 80 mg once daily. The patient clinically improved, so prednisolone was decreased to 40 mg, and the patient stayed on this dose throughout her hospital admission. For discharge, the patient was put on the following weaning plan as per dermatology advice. 40 mg once a day for two days, 35 mg once a day for seven days, 30 mg once a day for seven days, 25 mg once a day for seven days, 20 mg once a day for seven days, 15 mg once a day for seven days, 10 mg once a day for seven days, and 5 mg once a day for seven days and will be monitored closely in the clinic as an outpatient.

**Table 2:** Laboratory investigations at the point of deterioration

Investigation	Result	Reference ranges
White cell count	10.9	4-11 x 10⁹/L
Neutrophil count	8.91	1.5-7.5 x 10⁹/L
Eosinophil count	1.39	0.04-0.4 x 10⁹/L
Platelet count	65	140-400 x 10⁹/L
Lymphocyte count	0.33	1-4 x 10⁹/L
Alanine Transaminase (ALT)	303	10-49 U/L
Bilirubin	15	0-21 mcmol/L
Creatinine	128	60-120 mcmol/L
C-Reactive Protein (CRP)	41	0-10 mg/L

## Discussion

A number of studies and case reports have described chemotherapy-induced DRESS syndrome, with the majority involving chemotherapy regimens for hematologic malignancies, such as lymphomas and leukemias. For example, Rochling et al. (2020) reported several cases of DRESS in patients treated with rituximab-based chemotherapy, highlighting the need for heightened suspicion when chemotherapy is administered to immunocompromised patients [6]. Bocquet et al. (1996) emphasized the importance of recognizing the latency period of 2-8 weeks between drug exposure and symptom onset, which can delay diagnosis and complicate treatment [7]. The timing in this case, where the symptoms appeared about 3 weeks after chemotherapy, is typical of DRESS. Pham et al. (2021) described a fatal case of DRESS following pola-R-CHOP chemotherapy, where the patient presented with a rapidly progressive rash, multi-organ failure, and eosinophilia. Despite aggressive corticosteroid treatment and antiviral therapy, the patient succumbed to complications. This case underscores the potentially fatal nature of DRESS in heavily immunosuppressed oncology patients and highlights the need for early recognition [8]. A study by Figueroa et al. (2015) described CMV reactivation complicating the presentation of DRESS syndrome, emphasizing the difficulty in distinguishing between the two, especially in immunocompromised patients undergoing chemotherapy [9]. This interaction highlights the multifactorial nature of the syndrome and the need for careful monitoring of both drug-induced hypersensitivity and infection-related complications.

Other case reports detail hypersensitivity reactions with overlapping features of DRESS in chemotherapy, such as hypersensitivity pneumonitis and cytokine release syndrome, reflecting the diverse immune perturbations caused by these drugs [10]. These cases emphasize the variability in clinical severity and the importance of a high index of suspicion. While chemotherapy-associated DRESS remains rare, recent literature increasingly reports cases linked to multi-agent chemotherapy regimens, particularly those including immunomodulatory and cytotoxic agents.

## Conclusions

DRESS syndrome should be considered in patients receiving chemotherapy, especially those on polypharmacy. Early recognition is critical, as it can mimic other conditions like infections or SJS/TEN. This case underscores the importance of considering drug-induced hypersensitivity reactions, particularly when a patient presents with a progressive rash, fever, eosinophilia, and organ involvement 2-8 weeks post-treatment. While elevated eosinophil counts are commonly seen in DRESS, they may not always be markedly high. In this case, the eosinophil count was not as elevated as typically seen in DRESS cases, but the clinical presentation and the temporal relationship with chemotherapy strongly supported the diagnosis. Clinicians should consider DRESS even with mild eosinophilia. Early discontinuation of the causative drug and initiation of steroids is key in managing DRESS. The patient's clinical improvement with the introduction of IV hydrocortisone and the eventual switch to oral prednisolone underscores the efficacy of steroid therapy in reducing inflammation and preventing multi-organ failure. However, the management must be adjusted in response to other complicating factors, such as CMV reactivation. After diagnosing DRESS syndrome and starting treatment, ongoing monitoring is essential. In this case, the patient's deteriorating condition necessitated adjusting steroid doses and careful monitoring for adverse effects.
